# No-Reference Objective Quality Metrics for 3D Point Clouds: A Review

**DOI:** 10.3390/s24227383

**Published:** 2024-11-19

**Authors:** Simone Porcu, Claudio Marche, Alessandro Floris

**Affiliations:** 1 Department of Electrical and Electronic Engineering (DIEE), University of Cagliari, 09123 Cagliari, Italy; simone.porcu@unica.it (S.P.); claudio.marche@unica.it (C.M.); 2 National Inter-University Consortium for Telecommunications (CNIT), University of Cagliari, 09123 Cagliari, Italy

**Keywords:** point cloud, quality of experience, no-reference metric, objective quality evaluation, 3D, projection-based metric, model-based metric

## Abstract

Three-dimensional (3D) applications lead the digital transition toward more immersive and interactive multimedia technologies. Point clouds (PCs) are a fundamental element in capturing and rendering 3D digital environments, but they present significant challenges due to the large amount of data typically needed to represent them. Although PC compression techniques can reduce the size of PCs, they introduce degradations that can negatively impact the PC’s quality and therefore the object representation’s accuracy. This trade-off between data size and PC quality highlights the critical importance of PC quality assessment (PCQA) techniques. In this article, we review the state-of-the-art no-reference (NR) objective quality metrics for PCs, which can accurately estimate the quality of generated and compressed PCs solely based on feature information extracted from the distorted PC. These characteristics make NR PCQA metrics particularly suitable in real-world application scenarios where the original PC data are unavailable for comparison, such as in streaming applications.

## 1. Introduction

In recent years, the digital landscape has significantly moved toward more immersive and interactive technologies. Three-dimensional applications are leading this digital transition by gathering increasing attention from both researchers and industry professionals. Indeed, 3D applications represent a paradigm change in how we perceive and interact with digital information by offering more natural and intuitive approaches to content engagement and immersive first-person experiences when interacting with multimedia content. The impact of this technology extends far beyond mere visual experience, by including various sectors, from entertainment and education to professional training and remote working. A comparative analysis of 2D and 3D communication and interaction has demonstrated that immersive 3D technology is superior in creating collaborative and communicative scenarios, highlighting its potential to revolutionize how we connect and work in digital spaces [1].

Point clouds (PCs) represent a major way to capture and render 3D digital environments. They consist of a collection of sparse points in the 3D space, each typically associated with attributes such as color and normal vectors. One of the key advantages of PCs is their ability to be recorded in real time using devices like Light Detection and Ranging (LiDAR) sensors. This capability makes PCs particularly valuable in applications requiring immediate 3D representation, such as augmented reality (AR) and virtual reality (VR) object streaming. Despite their advantages, PCs present significant challenges in terms of data management. Indeed, the high-fidelity representation of complex 3D scenes often requires an enormous number of points, resulting in large datasets, which causes storage and transmission issues, particularly in bandwidth-constrained environments or applications requiring real-time data transfer.

To address these challenges, compression techniques have become crucial in the PC processing pipeline. Compression algorithms aim to reduce the data size of PCs while preserving as much of the original information as possible. However, to achieve significant data reduction, effective compression techniques must be *lossy*. This means that some information is inevitably lost during the compression process, leading to various types of distortions in the reconstructed PC. As a result, the compressed and subsequently decompressed PC is typically an approximation of the original, with potential degradations in quality and accuracy. This trade-off between data size and representation quality highlights the critical importance of PC quality assessment (PCQA) techniques, which are designed to measure the level of quality in reconstructed PCs, providing feedback on the efficacy and efficiency of compression algorithms and the overall fidelity of the 3D representation. PCQA techniques are crucial for choosing compression parameters or evaluating the performance of different algorithms. In this article, we aim to provide a comprehensive overview of a subset of PCQA techniques: no-reference (NR) objective quality metrics. The reason is that these metrics are designed to accurately estimate the quality of generated and compressed PCs based solely on feature information extracted from the compressed PC itself, without access to the original, uncompressed data. These characteristics make NR PCQA metrics particularly suitable in real-world application scenarios where the original PC data may not be available for comparison, such as in streaming applications.

To the best of the authors’ knowledge, this is the first article reviewing NR PCQA metrics. The survey in [2] is dated (published in 2017) and superficially describes some of the traditional point-to-point and point-to-plane PCQA metrics. Instead, the systematic review in [3] is very recent (published in 2024), but it is more focused on providing an overview of current established approaches and open challenges for PCQA, without taking a look behind technical details and performance comparison of the existing approaches. Therefore, by providing a comprehensive overview and comparison of state-of-the-art (SOTA) NR-PCQA techniques, this article aims to inform researchers on how to investigate the complex landscape of no-reference modeling approaches for the objective assessment of PC quality. As 3D technologies continue to advance and find new applications across diverse domains, the ability to efficiently and accurately estimate PC quality will remain a critical factor in ensuring the successful adoption of these immersive technologies. This review first provides a general overview of PCQA techniques and the available PCQA datasets in the SOTA. Then, the different approaches employed by NR PCQA models are detailed and compared in terms of the achieved quality estimation performance to outline the advantages and drawbacks of each method and to point out future research directions.

The paper is structured as follows. In Section 2, the background on PCQA and an overview of existing PCQA datasets are provided. In Section 3, we review the SOTA projection-based, model-based, and hybrid NR PCQA metrics. Section 4 discusses and compares the reviewed NR PCQA algorithms, highlighting their adoption advantages and drawbacks. Finally, Section 5 concludes the paper.

## 2. Background

### 2.1. Point Cloud Quality Assessment

The possible distortions introduced by the compression, reconstruction, and network transmission of PCs may introduce visual quality impairments that can be assessed using PCQA techniques. PCQA can follow a subjective or objective approach. Subjective approaches require a set of people to evaluate the quality of the distorted PC, typically using a 5-level Likert scale from which the Mean Opinion Score (MOS) is derived. Section 2.2 provides an overview of the most relevant PCQA datasets including subjective ratings provided for several types of distorted PCs. While conducting a subjective assessment provides the quality as perceived by the users, i.e., the Quality of Experience (QoE) [4], this approach has several drawbacks, such as being time-consuming and costly, and it may introduce bias due to evaluation scale or previous user experience.

**Figure 1:**
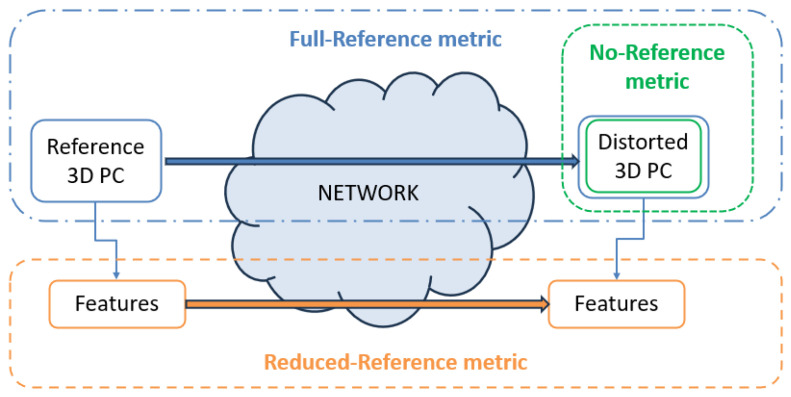
The scheme of full-reference (FR), reduced-reference (RR), and no-reference (NR) metrics.

Objective approaches have been developed based on mathematical algorithms to estimate the quality of the PC. According to the extent of reference PC data utilized to compute the quality metric, objective approaches can be categorized into full reference (FR), reduced reference (RR), and no reference (NR), which are illustrated in Figure 1. The FR needs the complete original and distorted PCs, the RR needs specific features extracted from the original and distorted PCs, while the NR only needs information from the distorted PC. Examples of SOTA FR approaches are GraphSIM [5], PC-SSIM [6], and PCQM [7]. GraphSIM [5] was designed to overcome the limitations of the earliest point-wise distance-based metrics adopted by MPEG, such as p2point [8], p2plane [9], and p2mesh [10]. Indeed, GraphSIM jointly considers the geometry and color distortion and uses the graph signal gradient as a quality index to evaluate PC distortions. PC-SSIM [6] explored the applicability of the Structural Similarity (SSIM) index (developed for images) in PCs, i.e., in a higher-dimensional, irregular space, including color and topological coherence among local regions. The authors investigated the effectiveness of various features derived from explicit and implicit information in a PC model and analyzed the impact of neighborhood size on quality scores. PCQM [7] is based on a weighted linear combination of geometry- and color-based features to estimate the quality of compressed PCs. Concerning RR approaches, the $PCM_{RR}$ metric [11] extracts a small set of statistical features from the reference PC in the geometry, color, and normal vector domains, which can be used to assess the quality of the distorted PC quality at the receiver side. The RR model proposed in [12] considers two features to evaluate the quality of PCs: the color fluctuation over a geometric distance and the color block mean variance. Although FR and RR models perform well for PCQA, they have the main drawback of requiring the complete reference PC or a part of it, respectively. Therefore, NR models are the most suitable approach for PCQA because they only need information from the distorted PC and thus can measure the PC quality in a real-time fashion. Moreover, NR has been demonstrated to achieve comparable or even increased quality estimation performance when compared with FR and RR models.

In this paper, we provide a review of the SOTA NR models for PCQA.

### 2.2. PCQA Datasets

This section provides an overview of the current PCQA datasets available in the SOTA, which are compared in Table 1 in terms of the number of reference and distorted samples and the number and type of considered distortion. PCQA datasets are essential to define novel objective models that can estimate the quality of distorted PCs as perceived by end-users. Thus, we focused on the datasets including a relevant number of distorted PCs and providing subjective ratings for each of them. The LS-PCQA contains the greatest number of distorted PCs and considers the most numerous and diverse quality impairments. The SIAT-PCQD is the only dataset whose PC qualities were evaluated using a head-mounted display (the HTC Vive), whereas all the others used a 2D screen.

**Figure 2:**
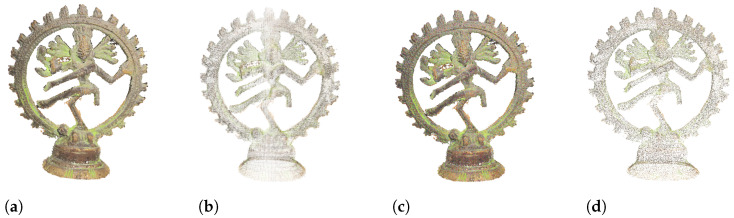
Examples of distorted PCs from the SJTU-PCQA dataset [13]. (**a**) Original Shiva PC. (**b**) OcTree-based compression (85%). (**c**) Color noise (70%). (**d**) Downscaling (90%).

The SJTU-PCQA (https://vision.nju.edu.cn/28/fd/c29466a469245/page.htm, accessed on 15 October 2024) dataset [13] provides 378 distorted PCs originating from 9 reference PCs. Seven types of common distortions corrupted each reference PC at six levels. Specifically, the considered distortions are OcTree-based compression, color noise, Geometry Gaussian Noise, down-sampling, the combination of down-sampling and color noise, the combination of down-sampling and Geometry Gaussian Noise, and the combination of color noise and Geometry Gaussian Noise. In Figure 2, we show one PC from this dataset (Shiva) impaired by three different distortions, namely OcTree-based compression, color noise, and downscaling.

The WPC (https://github.com/qdushl/Waterloo-Point-Cloud-Database, accessed on 15 October 2024) dataset [14,15] includes 20 high-quality reference PCs and 740 distorted PCs created using five distortions: downsampling, Gaussian noise, and three types of compression, i.e., G-PCC (Trisoup or Octree), and V-PCC. The same authors have provided a second version of WPC, i.e., the WPC2.0 (https://github.com/qdushl/Waterloo-Point-Cloud-Database-2.0, accessed on 15 October 2024) [12] dataset. This version includes 16 reference PCs from the WPC dataset (namely Bag, Banana, Biscuits, Cake, Cauliflower, Flowerpot, House, Litchi, Mushroom, Ping-pong bat, Puertea, Pumpkin, Ship, Statue, Stone, Toolbox), which were encoded using the MPEG V-PCC platform. Twenty-five distorted PCs were then created for each reference PC, for a total of 400 distorted PCs. Combinations of five geometry QPs (26, 32, 38, 44, and 50) and five color QPs (26, 32, 38, 44, and 50) were considered to create the distorted PCs. A third version, the WPC3.0 (https://github.com/qdushl/Waterloo-Point-Cloud-Database-3.0, accessed on 15 October 2024) dataset [16], is also provided, which consists of 350 distorted PCs. The 14 reference PCs were again selected from the WPC dataset (namely, Biscuits, Cake, Cauliflower, Litchi, Ping-pong bat, Puertea, Pumpkin, Ship, Statue, Toolbox, Coffeecup, Croissant, Saltbox, Honeydew melon) and encoded with V-PCC using the same values of geometry and color QPs used for WPC2.0.

**Figure 3:**
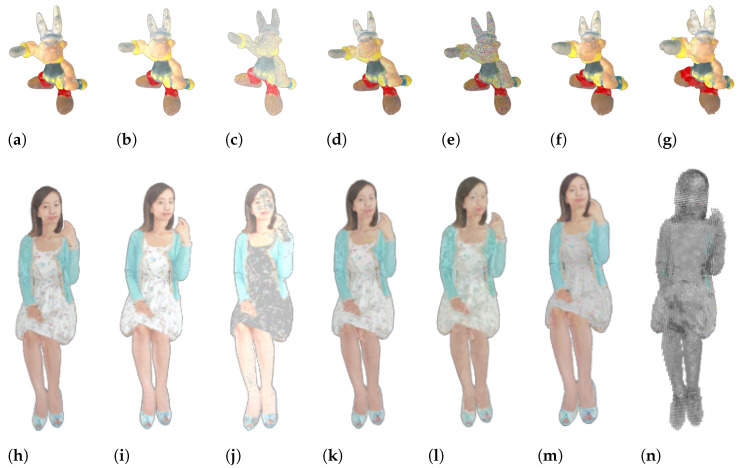
Examples of distorted PCs from the LS-PCQA dataset [19]. (**a**) Original Asterix PC. (**b**) Gamma noise with parameter 1. (**c**) Gamma noise with parameter 7. (**d**) Multiplicative Gaussian noise with parameter 1. (**e**) Multiplicative Gaussian noise with parameter 7. (**f**) Poisson Reconstruction with parameter 3. (**g**) Poisson Reconstruction with parameter 7. (**h**) Original Aya PC. (**i**) Poisson noise with parameter 3. (**j**) Poisson noise with parameter 7. (**k**) GPCC-Lossless geometry and lossy attributes with parameter 3. (**l**) GPCC-Lossless geometry and lossy attributes with parameter 7. (**m**) AVS-Limited lossy geometry and lossy attributes with parameter 3. (**n**) AVS-Limited lossy geometry and lossy attributes with parameter 7.

The SIAT-PCQD (https://ieee-dataport.org/documents/siat-pcqd-subjective-point-cloud-quality-database-6dof-head-mounted-display, accessed on 15 October 2024) dataset [17] comprises 20 reference PCs, distorted using 17 pairs of geometry and texture quantization parameters (QPs) with the V-PCC codec. In total, the dataset includes 340 distorted PCs. The M-PCCD (https://www.epfl.ch/labs/mmspg/downloads/quality-assessment-for-point-cloud-compression/, accessed on 15 October 2024) dataset [18] includes nine reference PCs, which have been compressed using five different encoders (namely, OctreeLifting, Octree-RAHT, TriSoup-Lifting, TriSoup-RAHT, and V-PCC) at five quality levels. The total number of distorted PCs is 225. Finally, the LS-PCQA (https://smt.sjtu.edu.cn/database/large-scale-point-cloud-quality-assessment-dataset-ls-pcqa/, accessed on 15 October 2024) [19] dataset contains 104 reference PCs, which were distorted with 31 types of impairments (such as Gaussian noise, contrast distortion, and local missing and compression loss) at seven distortion levels. The total number of distorted PCs is 22,568. In Figure 3, we show two PCs from this dataset (Asterix and Aya) impaired by different types and levels of distortions.

## 3. No-Reference PCQA Models

No-reference PCQA metrics are mathematical models that utilize information from the distorted PC received at the destination side to estimate the PC quality as perceived by the end user. To measure the accuracy of the quality estimation, this is typically compared with the mean subjective quality score (MOS) provided by a set of people during a subjective assessment. Common performance metrics are the Pearson linear correlation coefficient (PLCC), the Spearman’s rank correlation coefficient (SRCC), the Kendall rank correlation coefficient (KRCC), and the root mean square error (RMSE), which are all computed between the predicted and actual quality scores. The PLCC measures the linear correlation between the sets of actual and predicted quality data and can assume values between −1 and +1, indicating a positive and a negative linear correlation between the data sets, respectively. PLCC values around 0 mean the two data sets are not linearly correlated. The SRCC is a measure of rank correlation that assesses monotonic relationships between the two sets of data, i.e., whether the correlation between actual and predicted quality scores is linear or not. An SRCC of 0 means the data sets are not correlated, whereas SRCC values of −1 and +1 indicate perfect decreasing and increasing monotonic trends, respectively. The KLCC is also a measure of rank correlation, but it measures the ordinal association between the two data sets. Similar to PLCC and SRCC, the KLCC can assume values between −1 and +1. Finally, the RMSE is the quadratic mean of the differences between the actual and predicted quality values. The lower the RMSE, the higher the prediction accuracy of the quality model. Moreover, to be practically reliable, PCQA metrics need to deal with different kinds of distortions that may affect the PC during the compression, transmission, and reconstruction processes. Thus, most of the SOTA NR objective metrics are trained and validated on one or some of the datasets described in Section 2.2, which include an important number of PCs impaired by diverse distortions and accompanied by mean subjective scores.

**Figure 4:**
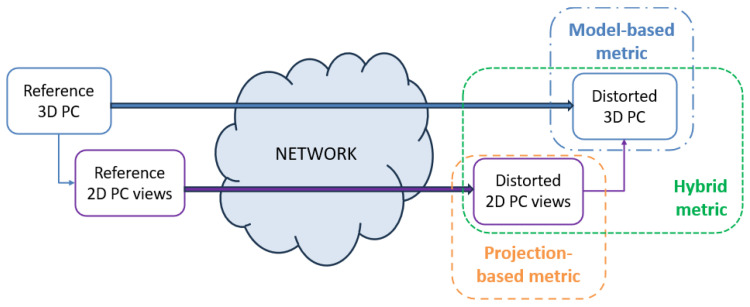
The scheme of model-based, projection-based and hybrid NR PCQA approaches.

NR PCQA approaches can be further classified into model-based and projection-based metrics based on the PC information considered for the quality estimate. Model-based approaches directly utilize the 3D PC to extract color and geometry information, which are used to identify and evaluate the impact of distortions on the PC quality. Model-based approaches are complex metrics, especially when dealing with sparse PCs where the point distribution may not adequately represent the underlying surface geometry. While dense PCs may contain millions of points, their regular distribution often simplifies the computation of quality metrics compared to sparse representations with fewer but irregularly distributed points. Thus, PC complexity is determined by the combination of the number of points and their spatial distribution (sparse or dense). Projection-based approaches rely on PC visualizations (2D projection of the PC) whose quality is analyzed and estimated using well-known techniques derived from SOTA images and/or video quality assessment algorithms or defining novel quality evaluation techniques, often based on deep learning. If information from both the 3D PC and 2D projections are used to extract data for quality estimation, the model follows a hybrid approach. Figure 4 illustrates the three approaches above.

The following sections review the most relevant SOTA studies proposing projection-based, model-based, and hybrid NR PCQA metrics.

### 3.1. Model-Based Approach

Model-based approaches extract color and geometry features from the 3D PC to estimate the quality of the PC. These models often make use of machine learning (such as in [20]) and deep learning architecture (such as in [19,21,22]), especially when dealing with sparse PCs composed of many points because computational complexity increases tremendously.

The ResSCNN model in [19] defines a sparse CNN architecture to extract the hierarchical features directly from 3D PCs. Indeed, the input of the hierarchical feature extraction module is a sparse tensor for PC, represented as a set of geometry coordinates and associated color features. In this study, the LS-PCQA dataset was also provided, which was used, together with the SJTU-PCQA and WPC2.0 datasets, to test the performance of the ResSCNN model. The PLCC between the actual and predicted quality of the distorted PCs achieved by ResSCNN is 0.60, 0.86, and 0.72 for the LS-PCQA, SJTU-PCQA, and WPC2.0 datasets, respectively. In [20], the 3D-NSS model is presented, which computes natural scene statistics (NSS) on different geometry and color features for the quality prediction of PCs. Geometry features include curvature, anisotropy, linearity, planarity, and sphericity, all calculated at the point level. The color features include the LAB color channels of each PC point. A set of statistical parameters (i.e., mean, standard deviation, entropy, generalized Gaussian distribution (GGD), asymmetric GGD (AGGD), and the shape-rate Gamma distribution) was then applied to the selected features to detect PC distortion. This set of features was used to train a support vector regressor (SVR) model, whose performance was validated on the SJTU and WPC databases. The 3D-NSS model achieved a PLCC of 0.7382 and 0.6514 and an RMSE of 1.7686 and 16.5716 on the SJTU and WPC databases, respectively. The ablation study shows that geometry features are major contributors to quality prediction, while color features and the application of statistics contribute to a lesser extent.

The graph convolutional PCQA network (GPA-Net) in [21] includes a new graph convolution kernel, i.e., GPAConv, which attentively captures the perturbation of the structure and texture of a 3D PC. Then, a multi-task decoder predicts the type and degree of the distortion and performs quality score regression. In the final step, coordinate normalization is performed to achieve the shift, scale, and rotation invariance that aims to stabilize the results of GPAConv. The PLCC between the actual and predicted quality of the distorted PCs achieved by GPA-Net is 0.886, 0.628, and 0.769 for the SJTU-PCQA, LS-PCQA, and WPC datasets, respectively. The model in [22], called GQI, considers geometric distance, mean curvature, and grey-level features from patches extracted around a set of PC points. The collected features are stacked and fed as input to a CNN model, which aggregates the predicted patch quality indexes to compute a global quality index for the PC. The PLCC achieved by the GQI-VGG19 (the model trained with VGG19 achieved the best performance) between the actual and predicted quality of the distorted PCs is 0.925 and 0.952 for the SJTU-PCQA and ICIP20 datasets, respectively.

The performance results achieved by these models evidence the superiority of deep learning architectures in identifying patterns and relationships between the color and geometry features extracted from the PC and the assessed PC quality. However, the drawback is that it is not easy to understand precisely what input–output relationships are established by the deep neural networks, in particular when they are composed of different layers and complex learning functions. Table 2 compares the reviewed model-based metrics in terms of considered distortion, used geometry and color features, considered feature statistics, and proposed quality prediction model.

### 3.2. Projection-Based Approach

Projection-based methods perform PC quality assessment based on the quality of PC 2D projections. The reference encoder for PCs is the MPEG V-PCC (video-based point cloud compression), which achieved the best performance in a call for proposals on PCC in 2017 [23]. The V-PCC encoding utilizes orthogonal projection onto a 2D grid to divide a PC into a set of patches, which are then merged into two separate video sequences containing the geometry and the texture information, respectively. The resulting video sequences can be compressed using traditional video compression techniques, which provide high compression ratios while maintaining the same visual quality. The majority of SOTA projection-based studies estimate the quality of PCs encoded using different values of geometry and texture QPs with the V-PCC algorithm. To build the prediction model, deep learning architectures (e.g., convolutional neural networks (CNN)) are widely used to extract features from 2D PC projections and find correlation with PC quality variations [24,25,26,27]. However, machine learning algorithms [28,29] and mathematical models were also considered [16,30]. Finally, the performance evaluation of the proposed models is performed on subjective ratings achieved from public PC datasets [16,24,25] or ad hoc subjective assessment results [27,29,30].

The IT-PCQA model, presented in [26], utilizes the rich prior knowledge in natural images to build a bridge between 2D and 3D quality assessment. First, IT-PCQA adopts a six-perpendicular-projection approach to create images from the PCs. These 2D images are fed into a Hierarchical Shallow CNN (H-SCNN), which extracts features from four different layers and combines them into a concatenated feature vector through average pooling. Then, a conditional-discriminative network employs adversarial domain adaptation to distinguish whether the features come from natural images or PCs. Finally, a quality regression network converts these features into objective quality scores. A Conditional Cross-Entropy Loss (CCEL) is introduced to penalize features that contribute less to the quality regression, thus refining the features for better objective score prediction. IT-PCQA was trained on two datasets of natural images (TID2013 [31] and LIVE [32]) and tested on two PC datasets (SJTU-PCQA and WPC). IT-PCQA (trained on TID2013) achieved comparable PLCC on SJTU-PCQA and WPC, i.e., 0.5791 and 0.5491, respectively. On the other hand, when trained on LIVE, the model achieved a PLCC of 0.5662 on SJTU-PCQA and 0.3099 on WPC.

In [24], the authors designed a video capture framework that rotates a camera around the PC via four symmetric circular pathways to cover sufficient quality-aware content and viewpoint changes. Then, spatial- and temporal-related features are extracted from the captured videos using a 2D-CNN and a 3D-CNN, respectively. These features are then fused and inputted to a two-stage fully connected layer consisting of 128 and 1 neuron, respectively, for feature regression and quality prediction. The proposed model achieved a PLCC of 0.8702, 0.8001, and 0.7244 and an RMSE of 1.1012, 13.5578, and 0.5561 on the SJTU-PCQA, WPC, and LSPCQA-I datasets, respectively. The LSPCQA-I is a reduced version of LSPCQA made up of 930 distorted PCs generated with 5 distortion levels of 31 types of distortions. The MS-PCQE neural network, presented in [25], extracts PC quality information using advanced visualization technology, i.e., projection scales and viewport attributes. The MS-PCQE consists of two main components: a multi-focal length feature interaction module and a Dual-branch Vision Transformer. The former module analyzes features based on distortion visibility under different focal lengths of the projection image, while the latter further processes the features to enhance global visual information by introducing long-distance feature characterization and mask-aware attention in convolutional features. The performance of the MS-PCQE model was evaluated on the WPC, SJTU-PCQA, and SIAT-PCQD datasets, yielding a PLCC of 0.8747, 0.9326, and 0.7879, and an RMSE of 11.0276, 0.8241, and 0.0785, respectively.

In [27], the proposed deep neural network, called PQA-Net, is structured into three main modules. The first module uses a multi-view projection to capture six 2D images of the PC; the feature extraction is performed through four CNN blocks, progressively reducing the images’ spatial resolution. The final feature vector, a 384-dimensional representation, i.e., 64 features for each of the six projections, is shared with the other two modules. The second module classifies the distortion type using fully connected layers into one of the predefined distortion categories. The last module is responsible for predicting the quality score of the PC and processing the features through additional fully connected layers. The output of this module is then multiplied by the distortion probability vector, obtained from the second module, to compute the overall quality score for the PC. The PQA-Net was trained and tested on the Waterloo Point Cloud Sub-Dataset, a dataset of 7920 distorted PCs created by augmenting the WPC dataset. Moreover, additional experiments were conducted using other datasets, where the PQA-Net achieved a PLCC of 0.85, 0.58, and 0.60 on the SJTU-PCQA (only distorted PCs with individual distortion types), IRPC, and M-PCCD datasets. Among these four deep learning-based models, the MS-PCQE [25] achieved the best quality prediction performance followed by [24] and PQA-Net [27], with this last one limited to considering one distortion at a time. Being trained on image datasets, IT-PCQA achieved the lowest performance.

The bitstreamPCQ model in [16] is a bitstream-layer model for coding the distortion assessment of V-PCC encoded PCs. In particular, this model first estimates texture complexity (TC) from the texture QP (TQP) and texture bitrate per pixel (TBPP); then, it develops a geometry distortion model as a function of the geometry QP (GQP). The quality prediction of the PC is provided by a mathematical model combining the texture and geometry assessment models. The bitstreamPCQ model performance was tested on the WPC3.0 dataset, achieving a PLCC of 0.9057 and an RMSE of 8.9586 between the predicted quality and the MOS. This is the only model tested on this dataset, so it is not comparable with the other SOTA models.

The last three projection-based models considered in this review focused on the streaming of dynamic PCs. Regression algorithms were utilized to determine the relationship between PC quality and PC distortion parameters, including those related to PC streaming, such as bandwidth and frame rate [28,29,30]. In [28], a sigmoidal fitting of a linear regression (LR) model was performed using several NR metrics (i.e., bandwidth, blur, blur ratio, noise, noise ratio, blockiness, and spatial information) calibrated against an objective FR benchmark (VMAF). The different weights and parameters of the LR were determined based on the particular video class, which was evaluated through the k-nearest neighbors (KNN) algorithm. After performing the LR, the model applies a sigmoidal mapping to the output to better model the perceived quality. The sigmoidal fitting is carried out per cluster, meaning each video class has a different set of sigmoid parameters. For the training and quality evaluation, the proposal made use of the dataset in [33], obtained from [34] by expanding with additional conditions and video scenes. The complete dataset results in a collection of sixteen source videos, each representing a generated viewport of a scene containing four PC objects. These objects were encoded using the V-PCC encoder, with five different quality levels ranging from 2.4 Mb/s to 53.5 Mb/s. The videos were then streamed using the Dynamic Adaptive Streaming over HTTP (DASH) protocol, employing various bandwidth conditions, resolutions, buffer lengths, and allocation algorithms. The total number of distorted PC videos is 453. The achieved PLCC and RMSE between the predicted quality and the objective quality computed with VMAF were 0.977 and 0.077, respectively.

In [29], a Gradient Boost regression model is proposed, which predicts the quality of PC videos in terms of compression information (geometry and attribute QPs, occupancy precision), video frame rate, and viewing distance. The dataset used to train the model was derived from a subjective assessment where test participants were asked to rate the quality of PCs compressed with the V-PCC algorithm at five different quality and three frame rates, and watched at three different viewing distances. The reference PCs were the *Dancer* from the Owlii dataset [35] and the *Thaidancer* from the 8iVSLF dataset [34]. The model achieved an $R^{2}=0.9754$ and a MSE=0.0175 between the predicted quality and the MOS. In [30], the authors introduced a fine-tuned ITU-T P.1203 model designed for predicting the quality of streamed dynamic PCs. The rationale is that PC streaming shares some parameters that can be used in the ITU-T P.1203 [36] model, such as bitrate, framerate, stall events, and viewing distance. The dataset utilized in [37] was considered to train the model. It consists of four PC objects (Loot, LongDress, RedAndBlack, and Soldier) from the 8i Voxelized Full Bodies Database [34], which were encoded with MPEG V-PCC using three different pairs of geometry and texture QPs to achieve different quality levels of PC videos. Moreover, video quality switches (quality switches in the middle of the sequences) were introduced, and different viewing distances were considered. Indeed, the distorted dynamic PC videos were watched and rated using the AR HoloLens 2. The coefficients of the ITU P.1203 were recomputed by applying regression between the achieved MOS and the considered distortion parameters. The fine-tuned P.1203 achieved a PLCC and an RMSE of 0.958 and 0.813, respectively, outperforming those achieved by the standard P.1203 model [36], i.e., 0.918 and 0.887. Although these models achieved high prediction performance, no validation with subjective scores was performed in [28], and ad hoc datasets were used in [29,30], making them not comparable with other SOTA approaches.

Table 3 compares the reviewed projection-based metrics in terms of considered distortion, used features, and proposed quality prediction model.

### 3.3. Hybrid Approach

To the best of the authors’ knowledge, the model in [38], called MultiModal Point Cloud Quality Assessment (MM-PCQA), is the only one leveraging the advantages of both 3D PC and 2D projected image modalities. In particular, the PC is first divided into sub-models to represent local geometry distortions, such as point shift and down-sampling, using a PC encoder. Simultaneously, an image encoder is used to extract texture features from 2D-rendered image projections of the PC. Finally, a symmetric cross-modality attention module is employed to fuse multi-modal quality-aware information. The PLCC achieved by MM-PCQA between the actual and predicted quality of the distorted PCs is 0.9226, 0.8556, and 0.8024 for the SJTU-PCQA, WPC, and WPC2.0 datasets, respectively. RMSE values are 0.7716, 12.3506, and 13.4289, respectively.

## 4. Discussion

This section discusses the main advantages and disadvantages of the approaches analyzed in the previous section. Additionally, the analysis of their performance on the community’s most well-known and recognized datasets is provided.

Table 2 and Table 3 provide a comparison of NR model-based and projection-based approaches, respectively. Each metric is evaluated based on diverse aspects, such as the type of distortions it tested against, the features used (geometry, color, and feature statistics), and the model implemented for quality prediction. The tables illustrate a common aspect among the approaches is their focus on testing similar types of distortions, particularly compression, downsampling, and noise, both geometrical and related to the color. This reflects the fact that these distortions are prevalent in PC processing and transmission and are usually included in the datasets used for testing.

Concerning the quality model, the focus of these approaches varies, with some relying on deep learning techniques (e.g., CNNs) to extract features from image projections [24], while others prefer to establish correlations between geometrical and color features and the perceived image quality [22]. It is interesting to note that while some methods extract detailed geometric descriptors like curvature and planarity [20], others rely on simpler features, such as geometry and texture QPs [29]. Furthermore, the choice of quality models varies, with deep learning techniques like CNNs [26] or DNNs [27] in general being widely adopted in some approaches, while others, like [20,29], prefer more traditional machine learning models like SVR or gradient boosting for computational efficiency. Another difference is represented in the use of feature statistics, which were relegated only to the model-based approaches. Also, it is easy to note that projection-based approaches are more numerous, probably because estimating the PC quality from the 2D projections makes it possible to reuse all the well-established knowledge concerning image and video quality assessment research studies.

However, even if the features change among the various methods, it emerges that the approaches using directly projected image frames as input require a very deep neural network to extract the information. All the other methodologies are lighter because they already have meaningful, elaborated features. Moreover, the latter approaches can weigh the meaning of each feature, highlighting their contribution to predicting PC quality. For instance, in [29], the authors noticed that higher quantization levels and frame rates lead to a higher MOS. In [24], the authors demonstrated through an ablation study that increasing the angular separation between keyframes leads to decreased performances. Moreover, the viewpoint-max-distance-sampled 7th/8th/22nd/23rd key frame selections can help improve the performance since these viewpoints can cover more PC content.

### 4.1. Performance Comparison

With regard to performance comparison, only a few of the reviewed studies are fairly comparable because of the evaluation performed using different datasets as well as diverse training/validation processes. However, a reliable comparison has been carried out in [25], which has implemented six SOTA models and compared them using the same training/validation procedures on the same datasets. Specifically, the considered NR metrics are two model-based (3D-NSS [20] and ResSCNN [19]), three projection-based (IT-PCQA [26], PQA-Net [27], and MS-PCQE [25]), and the hybrid approach MM-PCQA [38]. In the next sections, we compare and discuss the performance of these models on five SOTA PC datasets: SJTU-PCQA, WPC, SIAT-PCQD, M-PCCD, and LS-PCQA.

#### 4.1.1. SJTU-PCQA Dataset

**Figure 5:**
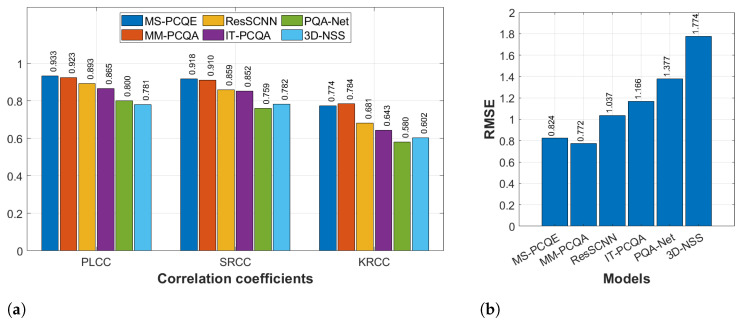
Performance comparison of six SOTA NR PCQA models on the SJTU-PCQA dataset as provided in [25]. (**a**) PLCC, SRCC, and KRCC. (**b**) RMSE.

Figure 5 compares the overall quality estimation performance of the six models on the SJTU-PCQA dataset in terms of PLCC, SRCC, KRCC, and RMSE. MS-PCQE slightly outperforms MM-PCQA in terms of PLCC (0.933 vs. 0.923) and SRCC (0.918 vs. 0.910), whereas MM-PCQA slightly outperforms MS-PCQE in terms of KRCC (0.784 vs. 0.774) and RMSE (0.772 vs. 0.824). The diverse types of distortions (compression, downsampling, geometry, and color noise) considered in this dataset are well captured by these two models compared to the others. However, good performance is also achieved by ResSCNN and IT-PCQA, while PQA-Net and 3D-NSS achieved the lowest performance results.

**Figure 6:**
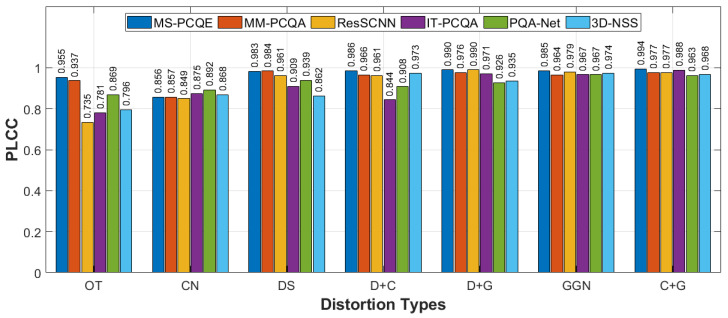
Performance comparison of six SOTA NR PCQA models, in terms of PLCC, on the various distortion types on the SJTU-PCQA dataset, as provided in [25].

**Figure 7:**
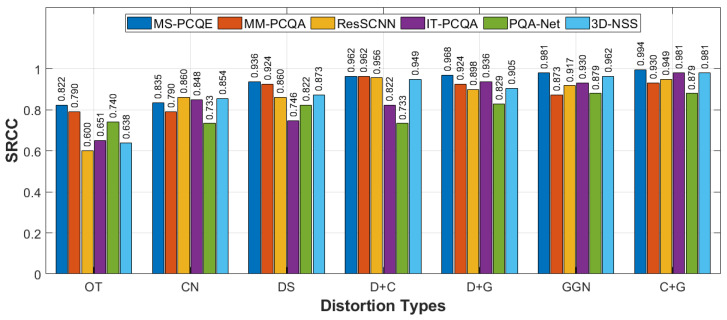
Performance comparison of six SOTA NR PCQA models, in terms of SRCC, on the various distortion types on the SJTU-PCQA dataset, as provided in [25].

Figure 6 and Figure 7 confirm these results by illustrating the performance of each model across the four individual distortion types considered in this dataset, i.e., OcTree-based compression (OT), color noise (CN), Geometry Gaussian Noise (GGN), and downsampling (DS), as well as three combined distortions: downsampling with color noise (D+C), downsampling with Geometry Gaussian Noise (D+G), and color noise with Geometry Gaussian Noise (C+G). MS-PCQE achieves the top performance in PLCC and SRCC across most distortion types, including OT, DS, D+G, and C+G, underscoring its capability to handle both single and combined distortions effectively. At the same time, MM-PCQA, a hybrid model, also performs consistently well, leveraging both 3D geometric and 2D projection features. ResSCNN shows strong results with specific distortions, particularly downsampling and geometry noise, due to its model-based focus on geometric fidelity. Still, it underperforms in scenarios involving subtle color degradations and compression, such as in the CN and OT categories. These findings emphasize the advantages of hybrid and projection-based models in handling diverse distortions. Hybrid models like MM-PCQA, which integrate both 3D geometric features and 2D projections, demonstrate the ability to address both spatial and visual quality, making them versatile in mixed-distortion cases. Projection-based models such as MS-PCQE show solid performance in perceptual distortions, capturing color and texture fidelity through multi-view simulations. In contrast, while effective in handling geometric distortions due to their focus on spatial features, model-based approaches tend to be less sensitive to perceptual factors like color variations and compression, limiting their adaptability in more complex distortion scenarios.

#### 4.1.2. WPC Dataset

**Figure 8:**
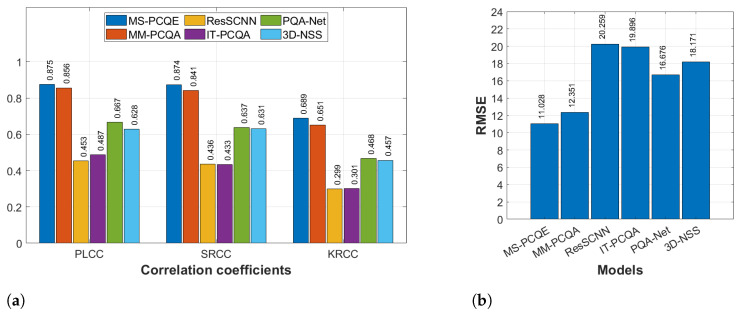
Performance comparison of most SOTA NR PCQA models on the WPC dataset as provided in [25]. (**a**) PLCC, SRCC, and KRCC. (**b**) RMSE.

Figure 8 compares the overall quality estimation performance of the six models on the WPC dataset in terms of PLCC, SRCC, KRCC, and RMSE. The MS-PCQE and MM-PCQA models again achieve the best performance (PLCC of 0.875 and 0.855, respectively), significantly outstripping all the other NR metrics. The third and fourth comparable best results are achieved by PQA-Net and 3D-NSS (PLCC of 0.667 and 0.628, respectively). The SRCC, KRCC, and RMSE results are consistent with the PLCC results. Thus, only the MS-PCQE and MM-PCQA metrics were demonstrated to be able to efficiently deal with the compression, noise, and downsampling distortions included in the WPC dataset.

**Figure 9:**
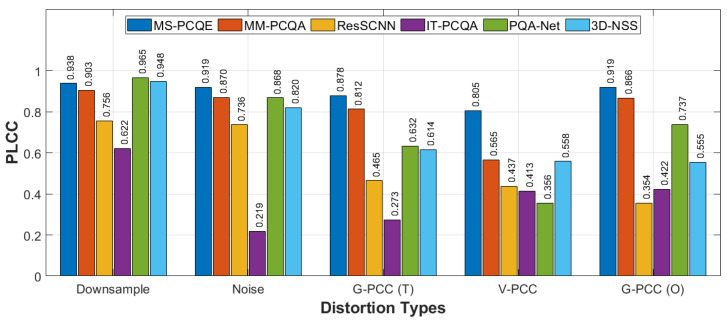
Performance comparison of most of the SOTA NR PCQA models, in terms of PLCC, on the various distortion types on the WPC dataset, as provided in [25].

**Figure 10:**
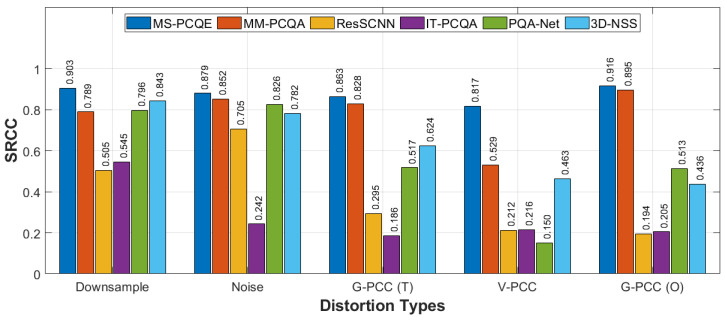
Performance comparison of most of the SOTA NR PCQA models, in terms of SRCC, on the various distortion types on the WPC dataset, as provided in [25].

Additional analysis on individual distortion types in the WPC dataset is shown in Figure 9 and Figure 10, including downsampling, Gaussian noise distortion, G-PCC Trisoup (T) compression, V-PCC compression, and G-PCC Octree (O) compression. The chart reveals that MS-PCQE performs well in handling downsampling and compression distortions, demonstrating particularly high values in both PLCC and SRCC. MM-PCQA, as a hybrid model, also demonstrates strong performance across the various distortions, except for V-PCC compression. PQA-Net and 3D-NSS achieved good results for downsampling and Gaussian noise distortions but fell short in compression distortions, particularly when color fidelity was impacted, as in G-PCC and V-PCC distortions. Similar trend results are achieved by ResSCNN, although with lower performance values. Finally, IT-PCQA did not achieve good performance for any distortion.

Similar to the findings described for the SJTU-PCQA dataset, these observations illustrate that projection-based and hybrid models have significant advantages over model-based approaches, especially in handling compression distortions. Projection-based models excel by simulating visual perspectives from multiple 2D views, making them particularly strong in assessing color and texture variations. Hybrid models, integrating geometric and visual cues, prove effective in addressing both spatial integrity and perceptual quality, adapting well to diverse and complex distortion environments. In contrast, model-based approaches focus on 3D geometric fidelity and spatial characteristics, providing reliable results for simpler geometric distortions but lacking sensitivity to color and texture variations introduced by compression processes.

#### 4.1.3. SIAT-PCQD

**Figure 11:**
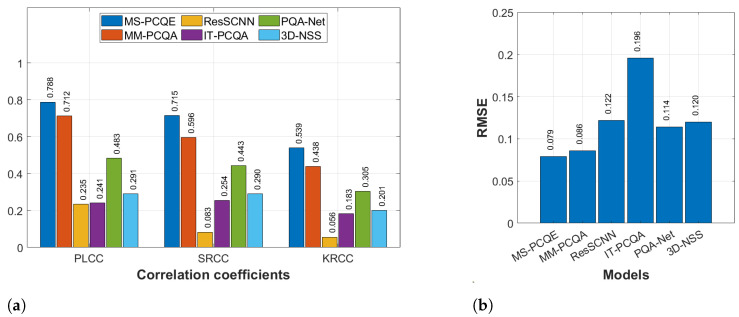
Performance comparison of most SOTA NR PCQA models on the SIAT-PCQD dataset as provided in [25]. (**a**) PLCC, SRCC, and KRCC. (**b**) RMSE.

Figure 11 illustrates the comparison regarding the SIAT-PCQD dataset, which considered compression distortions and is the only one including subjective scores provided using the head-mounted display. Even in this context, MS-PCQE shows the best performance (PLCC of 0.788) over the other methods, followed by MM-PCQA (PLCC of 0.712). All the other models achieved insufficient PLCC lower than 0.5, confirming the major difficulties of these models in dealing with compression distortions. Similar results were achieved for the SRCC and KRCC coefficients. On the other hand, IT-PCQA is the only model achieving an RMSE significantly greater than that achieved by the other models, whereas MS-PCQE and MM-PCQA still obtain the lowest estimation error. Since the SIAT-PCQD only includes compression distortions, these results may highlight the different quality perception of test participants watching distorted PCs on head-mounted displays rather than on 2D screens. Nevertheless, the distinction in model performance reveals that projection-based and hybrid models, such as MS-PCQE and MM-PCQA, respectively, maintain a significant edge over model-based approaches.

#### 4.1.4. M-PCCD and LS-PCQA Datasets

**Figure 12:**
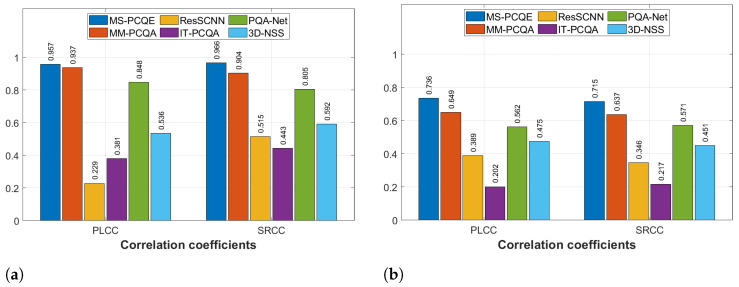
Performance comparison of most of the SOTA NR PCQA models in terms of PLCC and SRCC on the M-PCCD (**a**) and LS-PCQA (**b**) datasets as provided in [25].

Figure 12 depicts the quality estimation results on the M-PCCD (including only compression distortions) and LS-PCQA (including different types of distortions) datasets in terms of PLCC and SRCC. The MS-PCQE model continued to perform the best on both datasets (PLCC of 0.957 and 0.7356, respectively), always followed by MM-PCQA (PLCC of 0.937 and 0.649, respectively). PQA-Net is also confirmed in the third place for these two datasets (achieving acceptable performance at least for M-PCCD), followed by 3D-NSS. ResSCNN achieved the lowest performance on M-PCCD, while IT-PCQA performed the worst on LS-PCQA. In these two datasets, the advantage of MS-PCQE and MM-PCQA again underscores the strengths of projection-based and hybrid models, particularly when tested across diverse distortion types and intensities as those included in LS-PCQA. These results demonstrate that hybrid and projection-based approaches effectively address complex distortion cases and various PC content types, whereas model-based approaches may need further enhancement to adapt to the full range of distortions and higher complexity levels represented in these larger datasets. The lower performance achieved by MS-PCQE and MM-PCQA on the LS-PCQA highlights that there is a need to enhance the performance of NR metrics on different types and levels of PC distortion as well as on different types of PC contents. Indeed, the LS-PCQA includes the largest number of distorted PCs considering the largest number of distortions.

#### 4.1.5. Cross-Dataset Performance

Finally, Table 4 illustrates the cross-dataset performance of the two top-performing approaches, i.e., MM-PCQA and MS-PCQE. Specifically, as provided in [25], the table illustrates the cross-dataset analysis by training the two approaches on the LS-PCQA dataset, which contains the most diverse distortion types, and then evaluating their performance on the WPC and SJTU-PCQA datasets. The results show that MS-PCQE exhibits the best performance, confirming that the projection-based models currently perform the best compared to model-based or hybrid approaches even when evaluated on unseen data. However, MM-PCQA achieved almost comparable performance at least when validated on the WPC dataset.

### 4.2. Challenges and Future Directions

From the analysis of the performance of the SOTA NR PCQA models conducted in the previous section, we can identify the major challenges and future directions for improving the models’ performance. First, it can be noticed that the quality estimation performance achieved by these models is strictly related to the type of distortion impairing the PC. Specifically, most of the SOTA models achieved good performance on PCs distorted by Gaussian noise or downsampling distortions, whereas the major difficulties were encountered with color noise and compression distortions. The reason is mostly related to the features used to train the prediction model. As an example, IT-PCQA is trained on distorted image datasets, and it does not achieve good performance for distortions strictly related to PCs, such as those introduced by PC compression algorithms, which are not present in distorted images. Thus, deriving PCQA metrics from IQA datasets is quite limiting, although it can provide acceptable performance for distortions that are also present in distorted images, such as Gaussian noise and downsampling.

Second, the modeling approach is important. In particular, projection-based and hybrid approaches have been demonstrated to achieve greater performance than model-based approaches overall, particularly when dealing with compression distortions. Indeed, being typically based on geometry and color features, model-based approaches are more suitable when PCs are impaired by downsampling or noise distortions only, whereas they are not suitable for predicting the quality of compressed PCs. On the other hand, both projection-based and hybrid approaches achieved good to excellent performance on all types of distortions (except for IT-PCQA for the aforementioned reasons). The major strength of projection-based methods is to rely on different viewpoint projections and corresponding extracted features, which, processed by deep neural networks, enable the prediction model to deal with different types of distortions impairing the PCs. Additionally, hybrid approaches combine viewport information with geometry and color attributes extracted from the 3D PC. By including the strong points of model-based and projection-based approaches, hybrid models achieved state-of-the-art performance results.

Thus, future directions can be summarized as follows:

- The identification of features specifically related to PC compression distortions.
- Focusing on projection-based or hybrid-based approaches, which have been demonstrated to achieve the best performance. In particular, these models should consider multiview projections, mini-patch map sampling, and multiscale-based techniques, which have a relevant role in extracting features significant for quality estimation.
- Training the models on larger datasets including different types and levels of distortions. The lower performance achieved by all models on the LS-PCQA dataset is evidence that much still has to be carried out to enhance the robustness and generalization of models’ performance on larger datasets including diverse distortions.

## 5. Conclusions

The rapid advancements in recent technologies have underscored the reliance on PCs for real-time applications and underlined the importance of PCQA in ensuring that the reconstructed PCs’ quality remains acceptable despite the loss of information during compression. Unlike the FR or RR approaches, NR approaches have emerged as a practical solution in scenarios where uncompressed data are unavailable. We have reviewed the SOTA NR PCQA metrics by discussing the approach used (model-based, projection-based, and hybrid), distortions considered, features selected to estimate the quality, and the type of implemented model. We have compared the quality estimation performance achieved by the considered models, when possible, to discover the advantages and drawbacks of the diverse quality estimation approaches considered.

Our analysis showed that projection-based and hybrid models using deep learning architectures achieved the best quality estimation performance on the SOTA PCQA datasets. In particular, the MS-PCQE and MM-PCQA achieved the best performance on all kinds of considered PC distortions, which makes them the current reference models in the PCQA scenario. However, the lower performance results achieved by these models on datasets including a large number of distortions (LS-PCQA) and subjective scores provided using a head-mounted display rather than a 2D screen display (SIAT-PCQD) highlight that there are still open challenges that need to be addressed to develop an NR PCQA metric capable of performing the best in the majority of application scenarios.

## Figures and Tables

**Table 1:** Comparison of existing PCQA datasets.

Dataset	Number of Reference Samples	Number of Distortions	Number of Distorted Samples	Distortion Types
SJTU-PCQA [13]	9	7	378	Compression, downsampling, geometry noise, color noise.
WPC [14,15]	20	5	740	Compression, downsampling, noise.
WPC2.0 [12]	16	25	400	Compression.
WPC3.0 [16]	14	25	350	Compression.
SIAT-PCQD [17]	20	17	340	Compression.
M-PCCD [18]	9	25	225	Compression.
LS-PCQA [19]	104	31	22,568	Compression, color noise, geometry noise, downsampling.

**Table 2:** Comparison of SOTA NR model-based PCQA metrics.

Ref.	Distortion	Geometry Features	Color Features	Feature Statistics	Quality Prediction Model
[19]	Compression, downsampling, geometry noise, color noise	3D coordinates, occupation index	R, G, B color channels	-	Sparse CNN
[20]	Compression, downsampling, geometry noise, color noise	Point-level: curvature, anisotropy, linearity, planarity, and sphericity	L, A, B color channels	Mean, standard deviation, entropy, GGD, AGGD, Gamma	SVR
[21]	Compression, downsampling, geometry noise, color noise	PC structure	PC texture	-	Graph convolutional network
[22]	Compression, downsampling, geometry noise, color noise	Geometric distance, mean curvature	Gray-level features	-	CNN

**Table 3:** Comparison of SOTA NR projection-based PCQA metrics.

Ref.	Distortion	Features	Quality Prediction Model
[16]	Compression	Geometry QP, Texture QP, Texture bitrate per pixel (TBPP)	Mathematical Model
[24]	Compression, donwsampling, geometry noise, color noise	Video spatial and temporal information	CNN and DNN
[25]	Compression	Geometry QP, Texture QP	Dual-branch Transformer
[26]	Compression, downsampling, geometry noise, color noise	H-SCNN generated features	DNN
[27]	Compression, downsampling, geometry noise, color noise	CNN generated features	CNN and DNN
[28]	Compression, bandwidth, resolution, buffer length, allocation algorithm	Blockiness, noise, noise ratio, spatial information, and bandwidth, blur, blur ratio	KNN, LR and Sigmoidal Fitting
[29]	Compression, frame rate, viewing distance	Geometry and attribute QPs, occupancy precision, frame rate, and viewing distance	Gradient Boost Regression
[30]	Compression, quality switch, viewing distance	ITU-T P.1203 model features	Fine-tuned ITU-T P.1203 model

**Table 4:** Cross-dataset performance comparison, as provided in [25].

Model	LS-PCQA→WPC		LS-PCQA→SJTU-PCQA	
	PLCC	SRCC	PLCC	SRCC
MM-PCQA	0.6219	0.6149	0.7539	0.6987
MS-PCQE	**0.6653**	**0.6698**	**0.8210**	**0.7612**
